# Genome sequence of potential psychobiotic *Lacticaseibacillus rhamnosus* A20T1A using long-read ONT technology

**DOI:** 10.1128/mra.00685-24

**Published:** 2024-11-26

**Authors:** Irene Franciosa, Davide Buzzanca, Luca Cocolin, Francesca De Filippis, Ilario Ferrocino

**Affiliations:** 1 Department of Agricultural, Forest and Food Sciences, University of Torino, Grugliasco, Italy; 2 Division of Microbiology, Department of Agricultural Sciences, University of Naples Federico II, Portici, Italy; University of Maryland School of Medicine, Baltimore, Maryland, USA

**Keywords:** Oxford Nanopore technology, probiotics, psychobiotics

## Abstract

We present the genome sequence of *Lacticaseibacillus rhamnosus* isolated from human gut using Oxford Nanopore technology sequencing. The annotated draft genome of *L. rhamnosus* A20T1A showed the presence of probiotics and psychobiotics genomic traits relevant for gut colonization and human health.

## ANNOUNCEMENT

In the last years, lactic acid bacteria (LAB) have been increasingly reported to produce neuroactive molecules, able to modulate mood and cognition in humans (psychobiotics) (1). The strain was isolated from the human gut of healthy adult volunteers in Torino, Italy (Ethics statement Prot. no. 0676002) on da De Man, Rogosa e Sharpe agar (MRS, Neogen, USA; NCM0190A) incubated at 30°C for 48 h. Strain was purified on MRS broth under the same isolation condition and identified by Matrix-Assisted Laser Desorption Ionization-Time of Flight mass spectrometry (2). About 1 mL of the culture (on MRS broth at 30°C for 48 h) was then used for DNA extraction by the MasterPure Complete DNA and RNA Purification Kit (Biosearch Technologies). The strain was identified as *Lacticaseibacillus rhamnosus* sequencing was performed using the Rapid Sequencing Kit (SQK-RAD004, Oxford Nanopore, UK) and sequenced on a MinION Nanopore device R9.4.1 (FLO-MIN106, Oxford Nanopore, UK) according to the manufacturer instructions without DNA fragmenting, ligation, and size selection.

Default parameters were used for all software unless otherwise specified. 3.77 Gb of raw reads with a read length range from 1.38 to 26.88 kbp (average read length 3,360.92 bp) were subjected to base calling and low-quality base filtering with Guppy vr. 6.1.0 (3) and assembled using Flye v2.9.2 (--asm-coverage 50) (4). Overlap of the initial and final genome sequences was checked with BLAST+2.15.0 (5) showing one single circularized genome of 2.92 Mbp with a G + C content of 46.83%, genome coverage 100×. Annotation was performed by the NCBI Prokaryotic Genome Annotation Pipeline (PGAP v4.9) (6). The genome was then analyzed using *Tormes* v1.3.0 (7) that includes antibiotic resistance genes tools (ARG-ANNOT v.6, CARD v.2020, and ResFinder v.4) (8–10). The amino acidic sequences were analysed with e-mapper v2.1.12 (11) and Dfast v1.2.0 (12).

Genes annotation allowed the assignment of functional categories to 2,762 CDS, and the detection of 15 rRNA, 59 tRNA, and 2 CRISPR sequences. The most abundant genes were correlated to carbohydrate transport and metabolism, replication, recombination, and repair genes and amino acid transport and metabolism.

Part of the genes were putatively related to probiotic traits, that is, genes related to pilus structure (*spaE*, *spaD*, and *spaF*) and adhesion (*fbpA*). Beyond some antimicrobial peptides (AMP) gene related, that is, *ydeI* correlated gene (OmpD-associated protein), which confer AMP resistance via an unknown mechanism (13), we identified ABC transporters associated to bacteriocin export (14), and CAAX protease involved in bacteriocin self-immunity (15). Psychobiotics traits have been detected using BLASTn (−*e* value 1e−05) against psychobiotics genes selected from literature that identified correlations between these genes and probiotic and psychobiotic traits such as: tryptophan synthase (16), propionate synthase via fumarate hydratase (17, 18); indolelactate dehydrogenase (19), linoleate isomerase (20), genes involved in taurine ABC transport system (21), glutamate/gamma-amino butyrate antiporter (22) and genes involved in propionate synthase via malate dehydrogenase (18). This genome annotation allowed a better understanding of the psychobiotic traits of this strain.

### Nucleotide sequence accession number

*L. rhamnosus* A20T1A raw reads and assembly have been deposited to NCBI under accession numbers SRX22451036 and CP151435, respectively. FASTA file of psychobiotics genes has been deposited on ZENODO (https://doi.org/10.5281/zenodo.13923846).
